# Evaluation of the effects of the oxygen-inhibited layer on shear bond strength of two resin composites

**DOI:** 10.4103/0972-0707.48840

**Published:** 2008

**Authors:** Ankur Sehgal, Y Madhukar Rao, Martha Joshua, L. Lakshmi Narayanan

**Affiliations:** Department of Conservative and Endodontics, Saveetha Dental College and Hospitals, Chennai, India

**Keywords:** Composites, oxygen inhibited layer, shear bond strength

## Abstract

**Aim:**

The rising demand for aesthetic adhesive restorations has led to the wide use of composites. Multilayer techniques are recommended for the success of these restorations. However, this technique of layering causes the problem of interlayer adhesion, thus supporting the influence of the oxygen-inhibited layer. This study sought to test the hypothesis that the oxygen-inhibited layer increases the shear bond strength of composite resin by allowing the resins on both sides to cross the interface and form an interdiffusion zone.

**Materials and Methods:**

A microhybrid composite resin, Charisma, and a nanofill composite resin, Solare, were used in this study. Cylindrical specimens of the composites of 5 mm diameter and 6 mm height were prepared and embedded in acrylic resin moulds after curing. Curing was done in an argon atmosphere to prevent the formation of the oxygen-inhibited layer. To clinically simulate an inert atmosphere, a cellophane matrix strip was used during the process of curing.

**Results:**

Shear bond strength of the specimens was tested using a universal testing machine and the results were tabulated and statistically analyzed.

## INTRODUCTION

Typically, commercial dental composites are random copolymers of 2, 2-*bis* [4-(2-hydroxy-3-methacryloxypropoxy) phenyl] propane (*Bis*-GMA) and triethyleneglycol dimethacrylate (TEGDMA) and are filled with various types of inorganic particles. *Bis*-GMA and TEGDMA are bifunctional methacrylate monomers that harden after a free radical-induced polymerization reaction. This reaction is strongly inhibited by free radical scavengers such as oxygen.[1,2] The inhibition resulting from oxygen diffusing from the atmosphere into the curing resins is responsible for the formation of a soft, sticky, superficial layer commonly formed on freshly polymerized resins.[3–5] This is due to the oxidation of radicals into stable species known as peroxides, which have low reactivity towards monomers.[6,7]

$R^{*}+O_{2}\to R-{\mathrm{OO}}^{*}[\mathrm{stable radicals}]$

For years, the common perception was that the oxygen-inhibited layer is required for better bonding between the increments.

According to the principle of molecular interaction, the oxygen-inhibited layer should improve interfacial bonding. This layer not only adapts the overlying material to increase the contact area, but it also allows the materials on both sides to cross the interface and blend together to form an interdiffusion zone, where copolymerization can take place to produce a chemical bond.[8]

The aim of this study was to evaluate the effects of the oxygen-inhibited layer on the shear bond strength of two composites and test the hypothesis that the oxygen-inhibited layer increases bond strength.

## MATERIALS AND METHODS

### Resin composite and curing unit

The resin composites used in this study were Solare [GC] which is a nanocomposite and Charisma [heraeus kulzer] which is a microhybrid. The A2 shade of both the composites was used to reduce the effects of colorants.

The samples were polymerized using a Q Lux light curing unit [Dentsply] for 20 seconds per layer.

### Sample preparation

Sixty composite samples were prepared with standard dimensions of 5 mm diameter and 6 mm height. The samples were embedded in acrylic resin cylinders to facilitate testing in a universal testing machine.

Each sample consisted of two 3 mm layers each cured sequentially for 20 seconds.

The samples were divided into two groups:

Group I: consisted of 30 samples prepared using Solare composite resin, A2 shade.

Group II: consisted of 30 samples prepared using Charisma, A2 shade.

Each group was further subdivided into three subgroups of ten samples each.

Subgroup A: The samples were cured in an oxygen atmosphere.

Subgroup B: To avoid oxygen inhibition, the samples were cured in a glove bag filled with argon gas.

Subgroup C: To clinically simulate an inert atmosphere, a cellophane matrix was placed over the first increment and cured.

Following photopolymerization, all samples were conditioned at room temperature in deionized water for three hours.

### Shear strength measurement

The interlayer shear strength was determined using a Universal Testing Machine [Instron]. A shear force was applied to the interface with a crosshead speed of 0.5 mm/min.

The shear strength was calculated using the equation:

Shear strength = F/A

Where F = shear force at break point

A = π r^2^ = Cross-sectional area of interface

r = Radius of the sample

The average shear strength was calculated for each group. The data was analyzed statistically to determine significant differences between the groups. The mean and standard deviation (SD) values were estimated for the study groups and compared by one-way Anova and Tukeys-HSD test. Student's independent t-test was used to compare between the groups.

## RESULTS

The shear bond strength of samples in all subgroups was measured, and the results were statistically analyzed [Tables 1 and 2].

**Table 1:** Mean and standard deviation were estimated from the samples of each study group and compared by one-way Anova and Tukeys-HSD test

Mode of curing	Mode of fracture Group I	Mean SD value [Mpa]	Mean SD value Group II	*P*
Subgroup A	Cohesive	8.5 ± 0.2	6.6 ± 0.3	<0.0001
Subgroup B	Cohesive	7.4 ± 0.2	4.8 ± 1.7	
Subgroup C	Cohesive	6.2 ± 0.6	3.4 ± 0.3

**Table 2:** Student's independent t-test was used to compare between the groups

	*P* value	Inference
Subgroup A	< 0.0001	
Subgroup B	< 0.001	Group I > Group II
Subgroup C	< 0.0001	

## DISCUSSION

Dental composites are one of the most widely used restorative materials in dentistry. Multilayer techniques are recommended for the ultimate success of composite restorations to minimize the effect of polymerization shrinkage and to increase the degree of conversion. Bond strength between the different layers then becomes important. Hpwever, during the polymerization of the resins, diffusion of oxygen into the resin inhibits the polymerization reaction by forming peroxide radicals.[9] An unreacted double bond or a free monomer layer in the surface will remain after curing as the reactivity of oxygen is much higher with a radical than with a monomer.[10] This free monomer layer remaining on the surface after curing is known as the oxygen-inhibited layer and is always formed when the composites polymerize in the presence of air. A common perception is that the oxygen-inhibited layer is required before adding more layers of bonded composite to increase the strength. Reports on how this oxygen-inhibited layer affects the bond strength have been inconsistent. Studies have shown positive correlation indicating that the oxygen-inhibited layer increases the bond strength by the formation of covalent bonds within an interpenetrating network.[11,12] In contrast to this, it has also been reported that this layer induces brittleness due to inadequate links.[13,14] However, some recent studies have concluded that the presence of an oxygen-inhibited layer made no significant difference to the bond strength of composites.[3,8,15]

In the present study, shear bond strength of two composites (Charisma and Solare) was tested with and without the oxygen-inhibited layer. A microhybrid (Charisma) and a nanofiller (Solare) were used in this study as it has been found that interfacial strength decreases as filler loading changes from being highly filled to microfilled.[12] In agreement with this observation, this study found that Group I (Solare, a Nanofiller) showed higher bond strength than Group II (Charisma, a Microhybrid) although both had a cohesive type of fracture. Studies[16] have also shown that filler particles may act as obstacles to oxygen diffusion, may adsorb oxygen onto their surface, and allow oxygen diffusion along their surface. Increased oxygen solubility of the uncured resin due to adsorption of oxygen onto the surface of filler particles, may also provoke a decrease in conversion at the composite/atmosphere interphase.[17] This confirms that fillers may influence bond strength.

 Studies have shown that the absence of an oxygen-inhibited layer had no effect on the bond strength, and that bonding to a surface that was cured in an inert atmosphere (argon) generally gave higher strength.[18] Hence, argon atmosphere was used in one of the study groups. To clinically simulate an inert atmosphere, a cellophane matrix was used in the other group.

In the present study, shear bond strength values of composite cured with and without oxygen-inhibited layers were measured. The observation of a fractured surface is evidence that the majority of fractures occur at the substrate-specimen interphase and not at the interphase between the two increments of the composite samples which had an oxygen-inhibited layer. The influence of the oxygen-inhibited layer on adhesive strength may be because of the viscous nature of the unreacted methacrylate groups.[10] This layer not only readily adapts to the overlying material, but it also blends together to form an interdiffused zone, where copolymerization can take place to produce a chemical bond.[8] All these factors could strengthen the layer-layer interaction. The samples cured in the presence of argon and by using cellophane strips showed a cohesive fracture mode between the substrate and the layered composite, indicating that a strong bond was formed even without an oxygen-inhibited layer at the interphase. The results of the present study are in accordance with a previous study by Byoung[8] where he reported similar results. Taking these observations into consideration, it can be concluded that the presence or absence of the oxygen-inhibited layer does not have any influence on bond strength.

## CONCLUSION

Under the limitations of the present study and contrary to common perceptions, it can be concluded that the oxygen-inhibited layer is not necessary for bonding with the composite resin.
